# Identifying and reducing disparities in successful addiction treatment completion: testing the role of Medicaid payment acceptance

**DOI:** 10.1186/s13011-017-0113-6

**Published:** 2017-05-25

**Authors:** Erick G. Guerrero, Bryan R. Garner, Benjamin Cook, Yinfei Kong, William A. Vega, Lillian Gelberg

**Affiliations:** 1 Suzanne Dworak-Peck School of Social Work and Marshall School of Business, University of Southern California, 655 West 34th Street, Los Angeles, CA 90089 USA; 2 RTI, 3040 East Cornwallis Road, Durham, NC 27709 USA; 3 Department of Psychiatry, Harvard Medical School, 2 West Room 305, 401 Park Drive, Boston, MA 02215 USA; 4 Mihaylo College of Business and Economics, California State University, Fullerton, Fullerton, CA 90089 USA; 5 Department of Preventive Medicine, Keck School of Medicine, and Suzanne Dworak-Peck School of Social Work, University of Southern California, 655 West 34th Street, Los Angeles, CA 90089 USA; 6 Department of General Medicine, University of California, Los Angeles, Los Angeles, CA 90025 USA

**Keywords:** Disparities, Successful treatment completion, Racial and ethnic groups, Medicaid

## Abstract

**Background:**

Medicaid has become the largest payer of substance use disorder treatment and may enhance access to quality care and reduce disparities. We tested whether treatment programs’ acceptance of Medicaid payments was associated with reduced disparities between Mexican Americans and non-Latino Whites.

**Methods:**

We analyzed client and program data from 122 publicly funded treatment programs in 2010 and 112 programs in 2013. These data were merged with information regarding 15,412 adult clients from both periods, of whom we selected only Mexican Americans (*n* = 7130, 46.3%) and non-Latino Whites (*n* = 8282, 53.7%). We used multilevel logistic regression and variance decomposition to examine associations and underlying factors associated with Mexican American and White differences in treatment completion. Variables of interest included client demographics; drug use severity and mental health issues; and program license, accreditation, and acceptance of Medicaid payments.

**Results:**

Mexican Americans had lower odds of treatment completion (*OR* = 0.677; 95% CI = 0.534, 0.859) compared to non-Latino Whites. This disparity was explained in part by primary drug used, greater drug use severity, history of mental health disorders, and program acceptance of Medicaid payments. The interaction between Mexican Americans and acceptance of Medicaid was statistically significant (*OR* = 1.284; 95% CI = 1.008, 1.637).

**Conclusions:**

Findings highlighted key program and client drivers of this disparity and the promising role of program acceptance of Medicaid payment to eliminate disparities in treatment completion among Mexican Americans. Implications for health policy during the Trump Administration are discussed.

## Background

The current expansion of Medicaid in the United States to date has newly insured more than 16 million people and is playing an important role in reducing disparities in access to and engagement in care [1]. Insurance coverage is certainly the first step to reduce these disparities. But an often neglected factor that may contribute to disparities in access and engagement in care is provider acceptance of Medicaid [2]. Because the current health care policy environment requires evidence to revise the Affordable Care Act (ACA), it is critical to examine the role of Medicaid in eliminating health care disparities [1].

Regarding substance use disorder (SUD) treatment, much of the research on disparities has focused on differences between Whites and African Americans with regard to service access and use [3, 4], with only limited attention given to disparities in SUD treatment outcomes between non-Latino Whites and Latinos [5]. This represents an important gap in public health knowledge in an era of health care reform during which Latinos, particularly Mexican Americans, represent the largest population of uninsured individuals [6, 7] and most critically underserved ethnic minority group in the United States [8, 9]. Hence, we sought to identify individual and program characteristics associated with disparities in treatment completion.

This research is timely and can inform SUD treatment policy regarding the benefits and challenges of Medicaid as a mechanism to reduce the disparity gap in treatment completion. To support decision making related to the impact of Medicaid on quality of care, we sought to empirically assess the role of program acceptance Medicaid payment [10, 11] on reducing ethnic health care disparities, defined by the Institute of Medicine (recently renamed the National Academy of Medicine, or NAM) as all racial and ethnic differences except those due to clinical need, appropriateness, and patient preferences [12]. Establishing this relationship is important given three issues that challenge the health care system to improve quality of care for everyone: (a) the reduction or elimination of health-related disparities is a desired outcome in population health [1, 13–15] and would benefit any society; (b) empirical evidence regarding the impact of Medicaid expansion on health outcomes is extremely limited [16, 17]; and (c) lack of empirical evidence supporting Medicaid expansion is a barrier to justifying expansion efforts under the current administration [18].

In this study, we examined disparities and their drivers using rigorous statistical methods and critical theoretical frameworks. We relied on data from Los Angeles County’s multimillion-dollar SUD treatment outcome system [19] and followed Kilbourne and colleagues’ [20] three-phased disparities research framework, which includes a detection phase, understanding phase, and reduction phase. The purpose of the detection phase is to define health disparities, identify vulnerable populations, and develop valid measures for studying both. The purpose of the understanding phase is to identify factors that explain gaps in health and health care between vulnerable and less vulnerable groups, whereas the purpose of the reduction phase is to develop, implement, and/or evaluate interventions that may reduce or eliminate health and health care disparities. Consistent with this three-phased framework our three key research questions of interest were: Is there a disparity? What are the drivers of the disparity? Is Medicaid payment acceptance associated with reduction of the disparity? We completed the detection phase by examining the extent to which NAM-defined disparities exist between Mexican Americans and non-Latino Whites in terms of successful SUD treatment completion. We defined our outcome, successful treatment completion, as client report of sobriety at discharge, clinician report on clients’ alcohol- and drug-free status during the 30 days prior to discharge, and clinician decision to discharge clients successfully based on meeting treatment goals for that treatment episode. Second, we sought to complete the understanding phase by using a nonlinear adaptation of the Oaxaca–Blinder (OB) regression decomposition method [21, 22] to understand the factors underlying this disparity for Mexican Americans. This is a rigorous method to identify the extent to which differences between Mexican Americans and Whites in each of the covariates of interest explain the difference in treatment completion between these groups. Third, we sought to complete the reduction phase by testing the role of program acceptance of Medicaid payment in reducing the disparity by statistically testing differences in successful treatment completion between Mexican Americans and non-Latino Whites. This is the first study that relied on a large and unique multilevel dataset (programs and clients) to explore Mexican American disparities using advanced statistical methods, a framework to guide the disparities analysis, and theoretical frameworks to explain the client and program drivers of the disparity.

### Conceptual framework

We relied on sociocultural [23] and resource dependence [24] theoretical frameworks to explain program and client factors associated with outcomes. Racial and ethnic disparities in service use are driven by racial and ethnic differences in both health care system factors (e.g., policy, provider organization, provider factors) and community system factors (e.g., social context, social cohesion, and patient factors) that accumulate during the course of an individual’s illness. Stratified conditions are created when in the lower strata, health care markets fail, differential pathways into treatment develop, and there is poor patient–provider communication, lack of trust, and poor workforce availability or competence. As a result, racial and ethnic minorities have a greater risk than non-Latino Whites of dropping out of care and receiving lower quality of care, resulting in worse treatment outcomes [23, 25]. Thus in Hypothesis 1 regarding the detection phase, we posited that in both waves, after adjustment for clinical appropriateness and need, Mexican Americans will have lower rates of substance use treatment completion than non-Latino Whites.

Although the mechanisms of how these individual factors may inhibit Mexican Americans from successfully engaging in recovery have not been fully explored [5, 26], some empirical findings have suggested that these individual factors are negatively associated with treatment retention and posttreatment sobriety or abstinence [3, 5, 26]. In particular, disparate findings have suggested that these individual factors may create barriers to engaging fully in treatment and achieving sobriety or abstinence. Thus, in Hypothesis 2 regarding the understanding phase, we posited that the disparity will be driven by differences in individuals’ drug use severity (number of days of use during the past 30 days at program intake), psychosocial stressors (i.e., history of mental health disorders), and program characteristics (e.g., licensing and accreditation).

Additionally, Latinos, in particular Mexican-Americans are most likely to access publicly funded SUD treatment programs with low quality of care and limited service resources [4, 5]. Access to funding and technical support is critical for programs to improve quality of care, particularly among small and outpatient community-based treatment providers, which constitute more than 70% of the SUD treatment system [27–29]. SUD treatment organizations rely heavily on their regulatory and funding environment for financial and nonfinancial (i.e., professional expertise) resources, making them vulnerable to funding and regulatory expectations [30–32]. This is consistent with resource-dependence theory, which posits that high dependence on necessary resources determines an organization’s priorities to respond to key stakeholders [24]. By accepting Medicaid payments, programs strategically increase their revenue due to an increased number of clients with Medicaid. However, accepting Medicaid payments also pressures programs to be accountable for positive client outcomes. Because the most promising program interventions emphasize the importance of Medicaid for guaranteeing access to and retention in behavioral health services among low-income Latino clients [2, 33, 34], Medicaid acceptance may potentially reduce outcome disparities. Medicaid payment acceptance is associated with Latinos’ higher access to addiction treatment [35, 36], and for Mexican Americans this may lead to having the financial support to remain in treatment long enough to successfully complete treatment. Therefore, Medicaid payment acceptance may be especially beneficial for Mexican Americans by reducing treatment completion disparities; this will be assessed in moderation analyses by testing the significance of the coefficient for the Medicaid and Mexican American interaction term. Thus in Hypothesis 3 regarding the reduction phase, we posited that program acceptance of Medicaid payment will significantly reduce treatment disparities among Mexican Americans compared to programs not accepting Medicaid payments and non-Latino Whites.

## Methods

### Sampling frame and data collection

This study used a fully concatenated program and client dataset collected at two time points, 2010 and 2013. The sampling frame for program and client data included all SUD treatment programs funded by the Department of Public Health in Los Angeles County, California. Client data from these programs were drawn from the Los Angeles County Participant Reporting System, which includes standardized scales and questions related to client admission, discharge, and health derived from state (California Outcome Measure System) and federal (Treatment Episode Data Set) measurement systems [19]. Of approximately 14,000 treatment episodes involving clients from all racial and ethnic minority groups each year, client data were restricted to non-Latino Whites (38%) and Mexican Americans (32%). The final sample featured data from 7305 client treatment episodes collected from January 1, 2010, to December 30, 2010, and 8107 client treatment episodes collected from January 1, 2013, to December 30, 2013. The average age of clients in our sample was 36 years and 63% were men; 53.7% were non-Latino Whites and 46.3% were Mexican Americans. See Table 1 for descriptive statistics.

**Table 1:** Program and client characteristics reported as count (percentage) or mean (standard deviation)

	Wave 1 (2011)		Wave 2 (2013)		
	(*N* = 7305)		(*N* = 8107)		
	White	Mexican American	White	Mexican American	*p* ^a^
Client variables	(*n* = 4050)	(*n* = 3255)	(*n* = 4232)	(*n* = 3875)	
Treatment completion, *n* (%)*	749 (18.5)	695 (21.4)	483 (11.5)	589 (15.5)	< .001
Female, *n* (%)*	1529 (37.8)	1147 (35.3)	1666 (39.4)	1384 (36.0)	.166
Age, *M* (*SD*)	38.1 (12.9)	34.4 (11.7)	38.8 (13.1)	34.9 (12.3)	.011
Education, *n* (%)*					< .001
Less than high school	150 (3.7)	284 (8.7)	121 (2.9)	299 (7.8)	
High school	2647 (65.4)	2612 (80.3)	680 (16.1)	1676 (43.5)	
College	1174 (29.0)	347 (10.7)	2077 (49.1)	1497 (38.8)	
Postgraduate	79 (2.0)	12 (0.4)	1354 (32.0)	385 (10.0)	
Primary drug, *n* (%)*					< .001
Heroin	1251 (30.9)	652 (20.0)	1337 (31.6)	886 (23.0)	
Alcohol	1144 (28.3)	678 (20.8)	1157 (27.3)	653 (16.39)	
Methamphetamine	737 (18.2)	1111 (34.1)	918 (21.7)	1401 (36.3)	
Marijuana or hashish	288 (7.1)	478 (14.7)	213 (5.0)	635 (16.5)	
Other	630 (15.6)	336 (10.3)	607 (14.3)	282 (7.3)	
Days used, *M* (*SD*)*^b^	16.0 (13.0)	11.1 (12.8)	18.1 (12.8)	12.5 (13.1)	< .001
Age at first use, *M* (*SD*)*	20.7 (8.8)	19.3 (7.4)	20.6 (8.6)	19.1 (7.2)	.107
Medicaid eligible, *n* (%)*	988 (24.4)	997 (30.6)	591 (14.0)	1186 (30.8)	< .001
Mental health disorder, *n* (%)*	1380 (34.1)	612 (18.8)	1754 (41.5)	741 (19.2)	< .001
Treatment type					< .001
Outpatient	1635 (40.4)	1983 (60.9)	1257 (29.7)	2011 (52.1)	
Methadone	162 (4.0)	159 (4.9)	226 (5.3)	301 (7.8)	
Residential	2253 (55.6)	1113 (34.2)	2749 (65.0)	1545 (40.1)	
Program variables	(*n* = 122)		(*n* = 112)		
Medicaid payment, *n* (%)	85 (70.8)		65 (62.5)		< .001
Licensed, *n* (%)	115 (85.0)		98 (95.1)		< .001
Accredited, *n* (%)^c^	20 (16.8)		25 (24.5)		< .001

Note: Percentages calculated after removing missing values

*Difference between ethnic groups within wave is statistically significant at *p* < .051

^a^Indicates statistical significance between waves

^b^During 30 days prior to admission

^c^Accreditation by the Joint Commission

These clients were drawn from a random sample of 147 publicly funded programs located in communities with a population of 40% or more Latino, primarily Mexican Americans or African American residents in Los Angeles County. Client data were merged with program survey data from program managers using program identification. The provider sample for 2010 consisted of 122 programs with full and verified information, whereas the 2013 data featured 112 programs. Sixty-one programs had data at both time points.

### Dependent variables

#### Successful SUD treatment completion

This outcome relied on three indicators based on official discharge codes indicating whether clients successfully completed the major goals set forth in their recovery plan for that episode and whether clients reported sobriety at discharge. This dichotomous measure was coded 1 if clients met the following criteria: (a) the client reported no days of alcohol or drug use during the 30 days prior to discharge, (b) the clinician reported client sobriety at discharge, and (c) the clinician coded treatment episode as successful based on the client meeting treatment goals for that episode. This measure of treatment completion is more comprehensive than recent regional [37] and national [38] studies used in several analyses [39, 40].

#### NAM framework-informed clinical appropriateness and service need

This set of variables featured drug use severity at program entry (30-day drug use at intake), primary drug used, number of prior SUD treatment episodes, age at first drug use, and categorical measures of whether clients reported a history of mental health disorders or experienced homelessness at intake.

#### Medicaid insurance eligibility

Clients and clinicians reported whether clients were eligible for Medicaid; these reports were obtained from admission data from the Los Angeles County Participant Reporting System in 2010 and 2013.

#### Mexican American

This categorical measure featured a dummy variable representing whether clients reported having a Mexican background regardless of generation in the United States (1 = *Mexican American*; 0 = *not Mexican American*), with non-Latino Whites, also referred here as Whites, as the referent.

#### Demographic covariates

These covariates included client age, gender, and education.

#### Program covariates

These covariates indicated (a) whether the program accepted Medicaid payment; (b) whether the program was part of a parent organization or a standalone program; (c) whether the program was licensed by the state; (d) percentage of public funding received in the previous fiscal year; and (e) percentage of staff with graduate degrees.

### Analytic strategy

The detection phase identified ethnic disparities in substance use treatment completion following a three-step process informed by the NAM definition of health care disparities [41–43]: (a) model estimation; (b) a rank-and-replace methodology that adjusts for variables related to clinical appropriateness and need; and (c) prediction of rates of successful treatment completion for each racial and ethnic group using coefficients from Step 1 and adjusted characteristics from Step 2. In Step 1, a multiple logistic regression model was fitted to estimate the independent correlates of treatment completion. The logistic regression results are reported using odds ratios (ORs) and 95% confidence intervals (CIs). In Step 2, we used the rank-and-replace adjustment approach to create a counterfactual population of Mexican Americans with the distribution of need variables for non-Latino Whites. Clinical appropriateness and need variables were adjusted and used to calculate the disparity, whereas other variables such as key program measures (e.g., license, accreditation, Medicaid payment acceptance) were treated as non-need-related system-level variables that were not adjusted and therefore did not influence the disparity calculation. For more details regarding this method, please refer to Cook et al. [42].

Finally, Step 3, the prediction of sobriety at treatment completion for each ethnic group, used coefficients from the original multivariate regression model (Step 1) and the adjusted need covariate values (Step 2). The mean of these predictions was subtracted from the mean of predictions for non-Latino White clients to estimate a metric value of disparity. Variance estimates accounted for both the complex sampling design and multiple imputation of missing data (less than 8%). Variance estimates for disparity comparisons were calculated using a bootstrap procedure [44].

The understanding phase examined the association of individual- and program-level factors with treatment outcome disparities. Using the Fairlie variance decomposition method for nonlinear models [21], an extension of the OB decomposition method [45, 46], we estimated how much of the total difference in treatment completion between the two ethnic groups could be accounted for by each of the independent variables, while holding constant the other independent variables [22]. These analyses accounted for the clustering of clients within treatment facilities, adjusting standard errors for the correlation among clients of the same facilities [4, 38].

The reduction phase used the aforementioned multilevel logistic regression analysis to examine whether Medicaid payment acceptance was differentially beneficial (and disparity reducing) for Mexican Americans compared to whites. We relied on the STROBE statement to report all manuscript items required in rigorous observational studies.

## Results

Table 1 shows different percentages of unadjusted successful treatment completion, comparing ethnic groups and waves. In both waves, Mexican Americans had higher unadjusted rates of completion than non-Latino Whites (21.4 vs. 18.5% in 2010 and 15.5% vs. 11.5% in 2013, respectively).

**Fig. 1:**
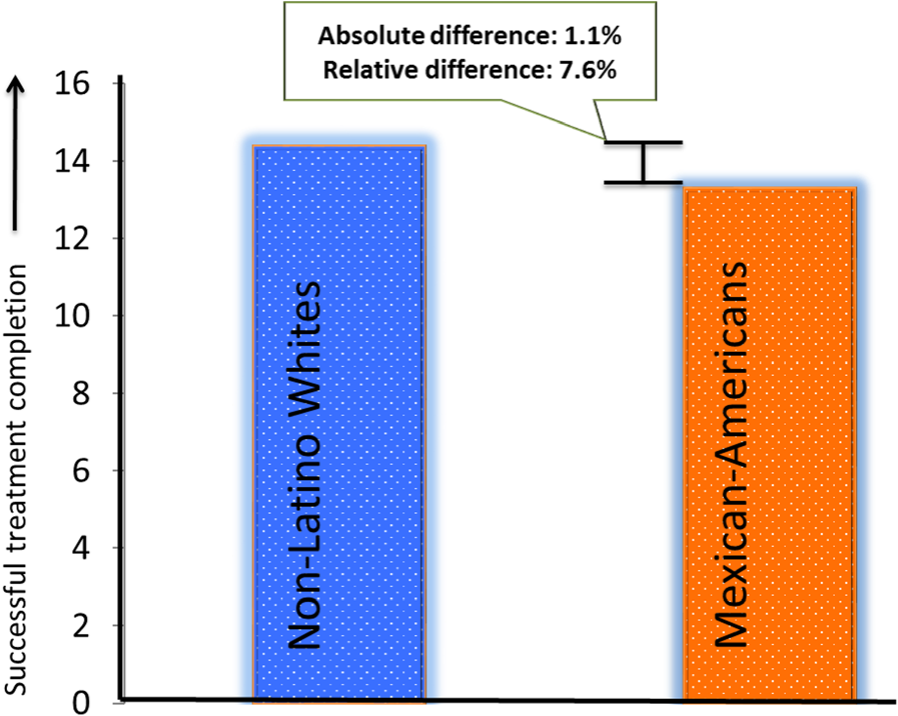
Disparity in successful treatment completion

Supporting Hypothesis 1, in the detection phase we found disparities in treatment completion in both waves after adjustment for clinical appropriateness and need, with Mexican Americans (13.3%) having lower rates of substance use treatment completion than non-Latino Whites (14.4%; *t*-test: Mexican Americans: *M* = 0.13, *SD* = 0.01 vs. Whites: *M* = 0.14, *SD* = 0.00, *p* < .001). The absolute difference is 1.1%, which corresponds to a relative decrease of 7.6% in the completion rate for Mexican Americans in relation to Whites. See Fig. 1. After further adjustment for the remaining individual socioeconomic and program factors in a multilevel logistic regression, compared to non-Latino Whites, Mexican Americans had significantly lower odds of treatment completion (*OR* = 0.677; 95% CI = 0.534, 0.859). See Table 2.

**Table 2:** Multilevel logistic regression of successful treatment completion

	OR	SE	95% CI	*p*
Program variables				
Wave 2^a^	0.479	0.062	0.372, 0.616	< .001
Medicaid payment	0.487	0.079	0.355, 0.668	< .001
Licensed	1.745	0.423	1.085, 2.806	.002
Accredited^b^	1.034	0.219	0.682, 1.567	.876
Cross-level interaction				
Wave × Mexican American	1.161	0.131	0.931, 1.447	.186
Medicaid × Mexican American	1.284	0.159	1.008, 1.637	.043
Client variables				
Mexican American	0.677	0.082	0.534, 0.859	.001
Female	0.932	0.075	0.796, 1.090	.379
Age	1.007	0.004	1.000, 1.014	.062
Education level	1.010	0.048	0.920, 1.108	.839
Primary drug^c^				
Alcohol	1.675	0.211	1.308, 2.145	< .001
Methamphetamine	1.777	0.321	1.247, 2.532	.001
Marijuana or hashish	1.689	0.252	1.260, 2.264	< .001
Other	1.744	0.221	1.361, 2.237	< .001
Days used^d^	0.954	0.010	0.936, 0.973	< .001
Age at first use	0.998	0.006	0.986, 1.011	.793
Medicaid eligibility	0.878	0.105	0.695, 1.111	.279
Mental health disorder	0.764	0.054	0.665, 0.878	< .001
Treatment type^e^				
Methadone	0.186	0.085	0.076, 0.456	< .001
Residential	0.791	0.148	0.548, 1.141	.209
No. of programs	143			
No. of clients	14,934			

Standard error values based on bootstrap method

^a^Wave 1 (2011) used as reference

^b^Accreditation by the Joint Commission

^c^Heroin used as reference

^d^During 30 days prior to admission

^e^Outpatient used as reference

Partial support for Hypothesis 2 was found. In the understanding phase, we posited that the disparity would be driven by differences in individuals’ drug use severity (number of days of use during the past 30 days at program intake), psychosocial stressors (i.e., history of mental health disorders), and program characteristics (e.g., Medicaid payment acceptance, licensing, and accreditation). The results of the variance decomposition analysis in Table 3 describe the contribution of each of the covariates to the unadjusted Mexican American-white difference in treatment completion. It is important to note that the Mexican American rate of treatment completion was higher than the White rate in the unadjusted comparison. Nonetheless, the decomposition allows for the identification of significant factors underlying differences between Mexican Americans and Whites. The O-B decomposition approach identified the contribution of each covariate to the unadjusted difference in mean treatment completion between Mexican Americans and Whites. Programs’ accepting Medicaid payments was a significant contributor to the unadjusted difference (*b* = 0.013; *SE* = 0.005). Other significant contributors were ethnic differences in rates of use of alcohol (*b* = −0.005; *SE* = 0.001); methamphetamine (*b* = 0.009; *SE* = 0.003); marijuana (*b* = 0.005; *SE* = 0.002); and other drugs (*b* = −0.003; *SE* = 0.001). See Table 3.

**Table 3:** Multilevel Oaxaca–blinder decomposition of differences for Mexican Americans and non-latino whites in treatment completion

	b	SE	95% CI	*p*
Overall				
Mexican American	0.175	0.022	0.132, 0.218	< .001
White	0.144	0.026	0.093, 0.196	< .001
Difference	0.031	0.021	−0.012, 0.073	.154
Explained	0.069	0.019	0.031, 0.106	< .001
Unexplained	−0.038	0.014	−0.066, −0.010	.009
Program variables				
Wave 2^a^	−0.002	0.002	−0.006, 0.003	.492
Medicaid payment	0.013	0.005	0.003, 0.023	.009
Licensed	0.000	0.001	−0.001, 0.002	.669
Accredited^b^	−0.001	0.006	−0.012, 0.010	.857
Cross-level interaction				
Wave × Mexican American	0.008	0.007	−0.005, 0.021	.230
Medicaid × Mexican American	0.015	0.008	−0.001, 0.032	.065
Client variables				
Female	0.000	0.000	0.000, 0.001	.458
Age	−0.003	0.002	−0.006, 0.001	.147
Education level	0.000	0.002	−0.005, 0.004	.843
Primary drug^c^				
Alcohol	−0.005	0.001	−0.008, −0.002	< .001
Methamphetamine	0.009	0.003	0.004, 0.015	.001
Marijuana or hashish	0.005	0.002	0.002, 0.008	.003
Other	−0.003	0.001	−0.005, −0.002	< .001
Days used^d^	0.025	0.010	0.006, 0.044	.012
Age at first use	0.000	0.001	−0.001, 0.002	.714
Medicaid eligibility	−0.001	0.002	−0.004, 0.002	.345
Mental health disorder	0.005	0.002	0.002, 0.009	.006
Treatment type^e^				
Methadone	−0.003	0.003	−0.009, 0.003	.296
Residential	0.005	0.004	−0.002, 0.013	.154

^a^Wave 1 (2011) used as reference

^b^Accreditation by the Joint Commission

^c^Heroin used as reference

^d^During 30 days prior to admission

^e^Outpatient used as reference

The 1.1% disparity is explained by differences in programs accepting Medicaid payments, professional accreditation, client Medicaid eligibility and treatment type, and differences unexplained by model covariates

Support for Hypothesis 3 was found. In the reduction phase, we posited that program acceptance of Medicaid payment would significantly reduce treatment disparities among Mexican Americans compared to programs that did not accept Medicaid payments and non-Latino Whites. The interaction between Mexican Americans and programs’ accepting Medicaid payment was statistically significant (*OR* = 1.284; 95% CI = 1.008, 1.637), meaning that improvements in treatment completion for those treated in programs accepting Medicaid were greater for Mexican Americans than Whites.

## Discussion

**Fig. 2:**
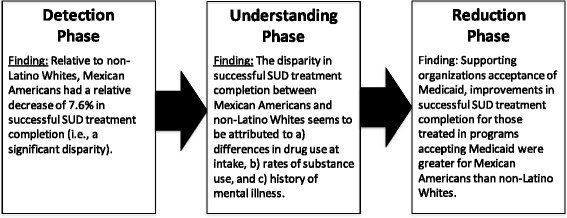
Findings using the three-phases of health disparities research from Kilbourne and colleagues [20]

As summarized in Fig. 2, which builds upon Kilbourne and colleagues’ [20] three-phased disparities research framework, the current study advances generalizable knowledge regarding three key questions. The extent to which disparities in successful SUD treatment completion exist between Mexican Americans and non-Latino Whites, which is the key identification phase question. The factors that explain disparities in SUD treatment completion between Mexican Americans and non-Latino Whites, which is the key understanding phase question. The extent to which variation in organization’s Medicaid acceptance is related to reductions in the successful SUD treatment completion disparity between Mexican Americans and non-Latino Whites, which is one of the key reduction phase questions.

For our identification phase question, analyses that were adjusted for clinical appropriateness and need in accordance with the NAM definition of health care disparity, identified a significant disparity (1.1% difference) between Mexican Americans and non-Latino Whites in successful SUD treatment completion. This finding is significant given that it helps address the paucity of research on Mexican American disparities, which has been identified by the NAM as a priority in terms of precisely distinguishing differences among Latino subgroups to address their specific needs [12].

In the understanding phase, the sociocultural [23] and resource dependence [24] theoretical frameworks guided our investigation of contributors to treatment completion differences. In this phase, the O-B decomposition identified how underlying differences in individual and program factors contribute to the overall difference. For example, clients’ primary drug of choice and programs’ Medicaid payment acceptance were significant contributors to differences between Mexican-Americans and non-Latino whites, whereas gender and age differences were negligible contributors. Specific findings of importance include: a) adjusting for Mexican Americans’ rates of substance use (alcohol + meth + marijuana + other drugs = −0.5% + 0.9% + 0.5%–0.3% = −0.6%) reduced Mexican American treatment completion rates and exacerbated the disparity by 0.9%, b) adjusting for Mexican American and White differences in days of drug use at program intake increased Mexican American treatment completion rates and reduced the disparity by 3.3%, and c) adjusting for Mexican American and White differences in history of mental illness increased Mexican American treatment completion rates and reduced the disparity by 0.5%. These findings are especially significant given that they highlight the critical importance of adjusting for clinical characteristics when considering how well a treatment system is supporting minority individuals in care.

Finally, for our reduction phase question, which focused on organizations acceptance of Medicaid payment, results indicated that this payment were associated with significant decreases in the disparity between Mexican Americans and non-Latino Whites regarding successful completion of SUD treatment. In other words, improvements in treatment completion for those treated in programs accepting Medicaid payment were greater for Mexican Americans than non-Latino Whites, suggesting that these programs were especially successful in assisting Mexican Americans in overcoming barriers to successful treatment completion during a significant period of time (2011–2013) in which expansion of Medicaid began to develop in California. This finding is of tremendous significance because it simultaneously advances Mexican American disparities research [5] and adds to emerging information on the impact of pre-Medicaid expansion on treatment outcomes [17, 41]. Furthermore, Los Angeles County will implement a comprehensive waiver program in July 2017 regardless of federal legislation on health care reform. Thus, the current findings support the waiver’s investment in Medi-Cal (California’s Medicaid program) for funding and service delivery regulation and individual characteristics of populations at higher risk of treatment dropout to improve treatment outcomes.

### Strengths and limitations

The main strength of this study is its reliance on unique and robust data from Mexican Americans and non-Latino Whites to identify the importance of adjusting for clinical characteristics to accurately identify and potentially reduce disparities. The large data on programs and clients drawn from a real-world health care system and the application of rigorous NAM and OB approaches added explanatory power to identify, understand, and reduce disparities.

However, the limitations of the study should be considered when interpreting results. In our disparities measurement, we did not adjust for patient preferences. Other studies have discussed the problematic elicitation of fully informed patient preferences. Nonetheless, to the extent that these preferences contributed to the disparity, our calculations were not completely concordant with the breadth of the NAM definition of health care disparity. Other measures (discrimination, structural barriers to completion, and organizational cultural competence) have been shown to contribute to health care disparities [3–5] and were unobserved in our data. Future studies should incorporate these variables if possible. Another limitation includes analyzing administrative client data and program survey data, but the accuracy and reliability of these data were enhanced by triangulating these data with observational data obtained during site visits. This resulted in dropping 5 % of programs with inconsistent information. Additionally, operationalization of success based on client self-reported 30-day alcohol- and drug-free status and clinician-reported client sobriety at successful treatment completion also could be improved. This outcome is also limited to a single treatment episode and does not consider that SUD treatment relies on a continuum-of-care approach. However, using two waves of data allowed us to provide robust results. Although the two-wave data did not allow us to establish causality, and differences in samples described in the Methods section may challenge the accuracy of changes reported at Wave 2, the sampling frame did not change and there were no statistically significant differences between and within programs in terms of reports of treatment completion. Finally, our analyses only allow us to generalize findings about service delivery to our sampling frame and not the wider addiction health services system. Nonetheless, this issue was somewhat mitigated by our large sample with two data collection time points of publicly funded SUD treatment programs serving communities with a population of 40% or more Latino, primarily Mexican-American or African American residents or both, representing approximately 7.7 million residents in Los Angeles County.

## Conclusion

The present study provided evidence supporting the relationship between a treatment program’s acceptance of Medicaid payments and treatment outcomes, especially in terms of having the potential to reduce important health disparities. Although further research is needed regarding both disparities for Mexican Americans and the impact of Medicaid on successful treatment outcomes, the present study nonetheless addressed significant gaps in the extant literature. This study provides evidence to support existing Medicaid coverage efforts, which again has been noted as a “critical piece of unfinished business” [1], and offers an opportunity to build on such efforts to promote health equity in California.

Because the ACA’s main principles of achieving universal health care and enhancing access to affordable and quality care for all Americans [14] are currently being debated under the current political administration, future research should explore how revised Medicaid coverage or other health insurance policies may affect the significant progress of reducing the uninsurance rate by 43% [1] and consequential effects on access to and benefit of high-quality care shown in some studies [6, 11, 36, 47]. In California, the new waiver program to be implemented in November 2017 will support the main principles of the ACA regarding access to care regardless of their potential federal repeal. It will be critical for researchers to track progress in treatment completion among the most vulnerable low-income and severely uninsured populations in California to inform national policy on the ultimate goal of improving public health for all.

## Abbreviations

ACA: Affordable care act
CI: Confidence interval
NAM: National academy of medicine
OB: Oaxaca-blinder variance decomposition
OR: Odds ratio
SUD: Substance use disorder
